# Forebrain neuroanatomy of the neonatal and juvenile dolphin (*T. truncatus* and *S. coeruloalba*)

**DOI:** 10.3389/fnana.2015.00140

**Published:** 2015-11-06

**Authors:** Roberta Parolisi, Antonella Peruffo, Silvia Messina, Mattia Panin, Stefano Montelli, Maristella Giurisato, Bruno Cozzi, Luca Bonfanti

**Affiliations:** ^1^Neuroscience Institute Cavalieri Ottolenghi, University of TurinOrbassano, Italy; ^2^Department of Veterinary Sciences, University of TurinTorino, Italy; ^3^Department of Comparative Biomedicine and Food Science, University of PadovaLegnaro, Italy

**Keywords:** bottlenose dolphin, striped dolphin, postnatal brain development, striatum, thalamus, cerebellum, lateral ventricle, germinative layers

## Abstract

Knowledge of dolphin functional neuroanatomy mostly derives from post-mortem studies and non-invasive approaches (i.e., magnetic resonance imaging), due to limitations in experimentation on cetaceans. As a consequence the availability of well-preserved tissues for histology is scarce, and detailed histological analyses are referred mainly to adults. Here we studied the neonatal/juvenile brain in two species of dolphins, the bottlenose dolphin (*Tursiops truncatus*) and the striped dolphin (*Stenella coeruleoalba*), with special reference to forebrain regions. We analyzed cell density in subcortical nuclei, white/gray matter ratio, and myelination in selected regions at different anterior–posterior levels of the whole dolphin brain at different ages, to better define forebrain neuroanatomy and the developmental stage of the dolphin brain around birth. The analyses were extended to the periventricular germinal layer and the cerebellum, whose delayed genesis of the granule cell layer is a hallmark of postnatal development in the mammalian nervous system. Our results establish an atlas of the young dolphin forebrain and, on the basis of occurrence/absence of delayed neurogenic layers, confirm the stage of advanced brain maturation in these animals with respect to most terrestrial mammals.

## Introduction

Experimentation on cetaceans is limited by obvious ethical concerns, which in several instances drastically reduce the availability of fresh tissue specimens. The peculiar anatomical conformation of whales and dolphins, and specifically the presence of a layer of blubber, greatly accelerate the post-mortem decay of nervous tissue upon stranding. Poor body conditions, and delay in samplings from stranded individuals, further contribute to the difficulty of obtaining quality samples for comparative neuroscience research, leaving several fine functional and neuroanatomical problems unsolved. Most of the studies on dolphin brain anatomy are restricted to adults (for general reference see Oelschläger and Oelschläger, 2009). The ubiquitous common bottlenose dolphin, *Tursiops truncatus* (*T. truncatus, Tt*), is the most extensively investigated cetacean species, yet there have been relatively few studies concerning its brain during fetal or early post-natal life. Studies on the development of the brain of dolphin species are scarce (Pirlot and Kamiya, 1975, 1982; Ridgway, 1986), and the majority of them were based on non-invasive approaches such as weight analysis at different ages (Marino, 1997), and magnetic resonance imaging (MRI) directed at analysing either the whole head (Liste et al., 2006; Rauschmann et al., 2006; Montie et al., 2007, 2008) or the whole brain (Marino et al., 2001b, 2004a) (see **Table 1**). These investigations provided anatomical correlates indicating that at birth the dolphin brain is already at an advanced stage of development (Ridgway, 1990), as observed for terrestrial Cetartiodactyla and hoofed animals in general (Peruffo and Cozzi, 2014). However, studies based on brain weight at different ages indicate that the brain of the bottlenose dolphin continues growing until the animal reaches adulthood (Ridgway and Brownson, 1984). The issue of mammalian brain development around birth and its subsequent maturation during the juvenile stages is important to fully understand to what extent and how long neural plasticity can persist in different species. Unlike laboratory rodents (that possess small lissencephalic brains and have a lifespan of 1–3 years), cetaceans and humans share several features including large brain size, advanced gyrification, long lifespan (see **Table 2**) and advanced cognitive performances.

**Table 1:** Literature available on the anatomical organization of the neonatal/juvenile dolphin brain.

Species	Age	Sex	Analysis	Aim of the study/conclusion	Reference
	*Newborn* (C.E.)	n.d.	CTI, FSh	Gross anatomy atlas	Liste et al., 2006
*Tursiops truncatus					
	*Postnatal* (<6 m)	M		MRI atlas	Marino et al., 2004a
		
	Fetus^§^ (6–9 m)	M			
				
	Fetus	M		Volumetric neuroimaging (WM/GM)	Montie et al., 2008
				
	*Neonate*	F			
*Lagenorhynchus acutus*			MRI		
	Subadult^§^ (2–3 y)	M			Montie et al., 2007^§^
				
	Subadult	M		MRI atlas^§^	
				
	Subadult	F			
		
*Delphinus delphis*	*Fetus* (8–9 m)	M		MRI atlas	Marino et al., 2001b

*Stenella attenuata*	*Perinatal*	n.d.	MRI, Gross dissection	Brain morphology and developmental stage	Rauschmann et al., 2006


*CE, Controlled environment; n.d., Not determined; MRI, magnetic resonance imaging; CTI, computed tomographic imaging; FSh, frozen sections of the head; WM/GM, white matter/gray matter volume ratio; Subadult, reproductively immature; Neonate, 126 to 140 cm. Comparable ages with respect to our analysis are in italics.*

*^§^Montie et al. (2007).*

**Table 2:** Key biological data of selected dolphin species.

Species	Pregnancy (months)	Newborn length (cm)	Adult length (cm)	Newborn brain weight (gr)	Adult brain weight (gr)	Sexual maturity (years)	Life expectancy (years)
*Tursiops truncatus*	12	100	200-300	676	1296–1930	5–10♀; 8–12♂	>50
*Stenella coeruleoalba*	12–13	90-95	200	430–460	785–980	5–13♀; 7–15♂	>58
*Stenella longirostris* (*orientalis*)	10	75–80	150–200	n.d.	660	4–7♀; 7–10♂	n.d.
*Stenella attenuata*	11.5	80–85	180–260	n.d.	n.d.	9–11♀; 12–15♂	n.d.
*Delphinus delphis*	11–12	80–90	200	476	750–875	6–7♀; 5–12♂	>30
*Lagenorhynchus acutus*	11–12	110–120	250–280	n.d.	1,200	6–12	>22


*White background: species analyzed in the article. Gray background: species used for comparison or quoted for reference; n.d., not determined. Biological data on *Tt*, *Sc*, and *Dd* are referred to Mediterranean specimens and taken from Cagnolaro et al. (2015); biological data on *Sl*, *Sa*, and *La* are referred to oceanic specimens and taken from Jefferson et al. (2015); brain weights of all species except *Stenella longirostris* are taken from Pilleri and Gihr (1970), and limited only to non-perfused brains of adult specimens (*Tt*: longer than 200 cm; *Sl* = *Stenella styx* and *Dd*:>170 cm). Brain weight of *Stenella longirostris* is taken from Hof et al. (2005). Weight of newborn brain of *Tt* and *Dd* are taken from Ridgway (1986); brain weight of newborn *Sc* is taken from Pirlot and Kamiya (1982) after authors’ correction for formalin fixation.*

Only a few studies have directly addressed the architectural and histological/immunocytochemical aspects of the dolphin brain (Hof et al., 2005; Furutani, 2008; Kern et al., 2011; Butti et al., 2014; Mortensen et al., 2014). We took advantage of a stock of cetacean brain material collected by the *Mediterranean marine mammal tissue bank* (MMMTB) of the University of Padova to study the neonatal/juvenile dolphin brain neuroanatomy and developmental stage. We analyzed brain tissues obtained from 13 specimens of two different species, namely the bottlenose dolphin and the striped dolphin *Stenella coeruleoalba* (*S. coeruleoalba, Sc*), at neonatal, juvenile, and adult ages. **Table 2** provides a synthetic comparison of the key biological parameters of the two species examined here (*Tt* and *Sc*), as well as four very similar species whose adult brains were been described by imaging methods (the short-beaked common dolphin *Delphinus delphis*, *Dd*; the [Eastern] spinner dolphin *S. longirostris* [*orientalis*], *Sl*; the pantropical spotted dolphin *S. attenuata*, *Sa*; and the Atlantic white-sided dolphin *Lagenorhynchus acutus*, *La*) or in a conventional atlas (*Dd*). We emphasize that all these species belong to the dolphin family and have closely related morphology. Furthermore, at least two of the dolphin species listed above (i.e., *Sc* and *Dd*) live in mixed schools (Bearzi et al., 2011) that may contain hybrids.

In the present article, we report data obtained at different ages, and compare the brain anatomy of very young (neonatal/juvenile) and adult dolphins, by combining histological studies, analysis of white/gray matter ratio (WM/GM), and of white matter myelination in selected regions. The data were also compared to those available in the literature for adult specimens (e.g., MRI descriptions) and to data obtained for mice (for cerebellum; see below). To better characterize the developmental stage reached by the central nervous system (CNS) of dolphins at birth, the analysis was extended to the cerebellum, to identify the existence/exhaustion of transient germinal layers in the cerebellar cortex, a hallmark for mammalian CNS development (Altman, 1969). In parallel, our study was aimed at obtaining a more detailed neuroanatomical definition of the neonatal/juvenile dolphin forebrain, which in terrestrial mammals is known to host the persistence of neurogenic processes in the form of stem cell niches lining the lateral ventricle wall (the so-called subventricular zone; see Bonfanti and Ponti, 2008; Bonfanti and Peretto, 2011).

## Material And Methods

### Tissue Samples

#### Dolphin Tissues

In this study we used samples from the brains of 13 dolphins, 10 bottlenose dolphins, and 3 striped dolphins, stored in the MMMTB of the University of Padova at Legnaro, Italy (see **Table 3**). The MMMTB is a CITES recognized (IT020) research center and tissue bank (Ballarin et al., 2005), sponsored by the Italian Ministry of the Environment and the University of Padova. The MMMTB includes tissues from (a) cetaceans stranded along the Italian coastline, and (b) captive dolphins sent to the Department of Comparative Biomedicine and Food Science of the University of Padova for post-mortem examination. Tissue samples preserved in the MMMTB are distributed to qualified research centers worldwide, following a specific documented request, according to national and international regulations on protected species (CITES).

**Table 3:** Detail of the sampled bottlenose and striped dolphins.

Specimen	ID	Sex	Origin	Length/Weight	Age
*T. truncatus*	186	F	C.E.	110,5 cm/19kg	9 days (neonatal)
	145	M	C.E.	118 cm/19kg	7 days (neonatal)
	144	M	C.E.	117 cm/22,1kg	9 days (neonatal)
	229	M	C.E.	137 cm/21,9 kg	9 days (neonatal)
	162	M	Stranded	119 cm/20,3 Kg	20 days (neonatal)
	344	M	Stranded	195 cm/98,5 Kg	Subadult
	192	F	Stranded	240 cm/178,5 kg	Adult
	196	M	Stranded	300 cm/219 kg	Adult
	203	M	Stranded	284 cm/288 kg	Adult
	319	M	Stranded	310 cm	Adult
*S. coeruleoalba*	320	F	Stranded	137 cm/21,9 kg	Juvenile (3–6 months^a^)
	167	M	Stranded	198 cm/94 kg	Adult
	218	M	Stranded	198 cm/81 kg	Adult


*CE, Controlled environment; ^a^estimated on the basis of the body weight and length.*

The bottlenose and the striped dolphin have very similar shape and anatomy; differences in size and weight (*Tt* is generally larger than *Sc*) are actually well evident in oceanic animals, but they are less marked in dolphins living in comparatively smaller basins (including the Mediterranean). Ages will be referred to as neonatal (shortly after birth; 7–20 days), juvenile (3–6 months), and adult (15–50 years). A synthesis of the key biological parameters of the two species is reported in **Table 2.**

**FIGURE 1:**
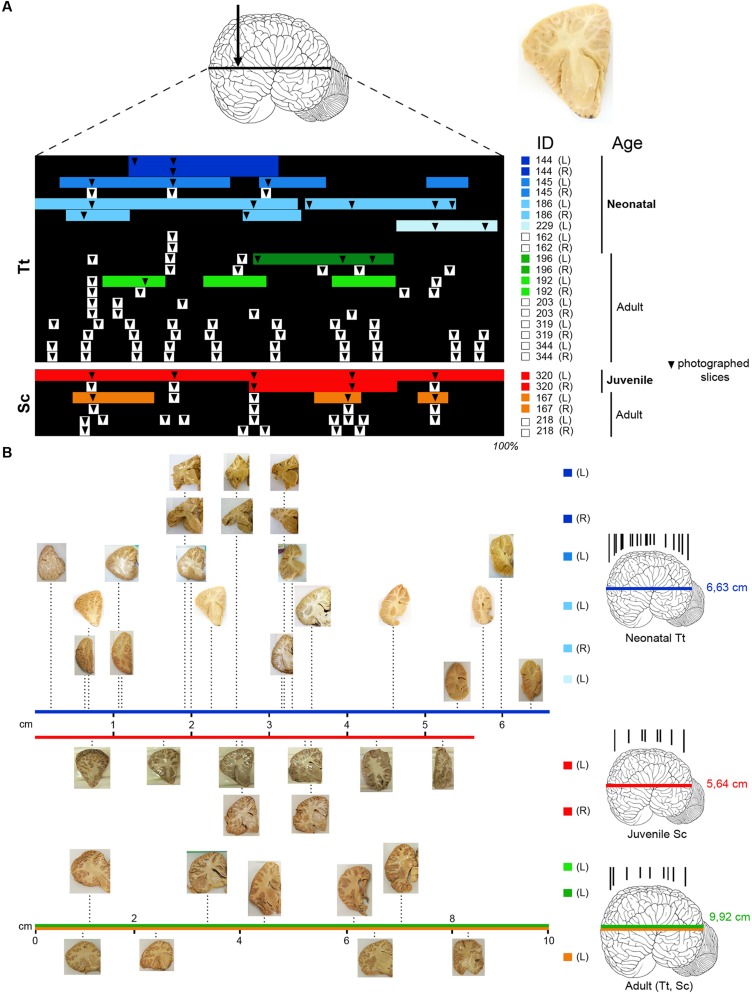
**(A)** Brain tissue samples of *Tt* and *Sc* used in this study. Arrow, orientation of coronal cuts performed to obtain thick brain slices (an example is showed on the right). ID, identification numbers of all animals used (see **Table 3**); L, left hemisphere; R, right hemisphere. Color lines indicate the amount of tissue analyzed histologically for each animal and hemisphere (neonatal *Tt*, shades of blue; juvenile *Sc*, red; adult *Tt*, shades of green; adult *Sc*, shades of yellow), as a percentage of the whole brain extension (black backlot; not in scale). Arrowheads indicate the position of slices photographed to establish anterior–posterior levels; those outside the color lines were used for quantification of white/gray matter ratio (white squares on the right indicate animals used only for these analyses). **(B)** Photographs of sample slices, positioned at their exact level along the brain anterior–posterior axis (levels in scale with respect to the whole brain length; total brain length – indicated on the right, beside the brains – not in scale); all slices are represented with the lateral ventricle on the right, with the same orientation used in the drawings in the following figures. On the right, representative brains showing the position of all coronal cuts performed to obtain the slices (in each age group brains from different animals are considered; not in scale). The length values are referred to Tt-186 (blue line), Sc-320 (red line); the value in adults (9,92 ± 2,6 cm) is an average length obtained from an estimation of our material and data reported in literature, for both *Tt* and *Sc* (green and yellow lines).

**FIGURE 2:**
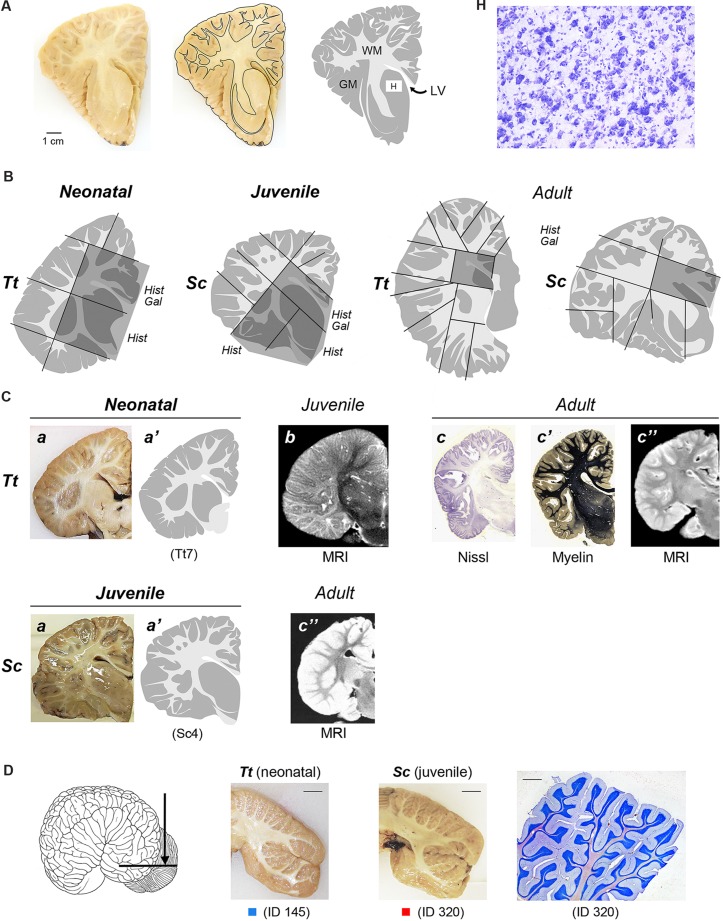
**(A)** To obtain a graphic representation of single brain slices the contours of each coronal section surface (external -pial- surface and white/gray matter limits) were drawn: GM, gray matter (gray areas); WM, white matter (white areas); LV lateral ventricle; H, histological analysis (Cresyl violet staining) was performed within some areas, with particular reference to the subcortical gray matter of forebrain regions (see also **Figures 4** and **5**). **(B)** Representative drawings showing how single, thick brain slices were cut into blocks (black lines) to be included and frozen for subsequent cryostat cutting; dashed areas indicate the blocks analyzed histologically in these representative slices; all blocks analyzed in this study at the different anterior–posterior levels are listed in **Table 4.** *Hist*, histological analysis; *Gal*, Gallyas method. **(C)** Comparison of drawings obtained from neonatal *Tt* and juvenile *Sc* samples with information available from literature, obtained with different approaches at different ages. a,a’, representative slices of neonatal *Tt* and juvenile *Sc* brain material analyzed in this study (see **Figure 1**); a, coronal brain slices; a’, drawing carried out at the same level; b, originally acquired coronal magnetic resonance images (MRIs) sections of the infant brain (less than 6 months; from Marino et al., 2004a). c, c’, c”, reference images of adult dolphin brains; originally acquired coronal sections stained for cell bodies with Nissl method (c) and for myelinated fibers (c’), from The Yakovlev-Haleem collection at the National Museum of Health and Medicine, Armed Forces Institute of Pathology; c” originally acquired coronal MRI sections of the adult brain (from Marino et al., 2001a – *Tursiops truncatus*; from Marino et al., 2004b – *Stenella longirostris orientalis*). **(D)** Cerebellar tissue samples used in this study. On the left, coronal cutting direction (arrow) to obtain thick slices. In the middle, two representative coronal slices from neonatal *Tt* and juvenile *Sc*; scale bars, 1 cm. On the right, cresyl violet staining of cerebellar lamellae; scale bar, 200 μm.

Tissue samples used for the present investigation consisted of coronal brain slices (**Figure 1**), including the cerebellum (**Figure 2C**), approximately 1–1.5 cm thick, collected in the necropsy room of the Department of Comparative Biomedicine and Food Science of the University of Padova at Legnaro, and fixed by immersion in 4% buffered formalin. Post-mortem delay before actual sampling varied between 18 and 40 h.

#### Mouse Tissues

All experimental procedures conducted according to the European Communities Council Directive and the Italian law for the care and use of experimental animals. Mouse tissues were obtained from CD-1 mice (Charles River, Italy) used in a previous work (Ponti et al., 2006b; refer to this publication for experimental procedures) and stored in our lab at the Neuroscience Institute Cavalieri Ottolenghi (N.I.C.O., Orbassano, Turin, Italy). Cerebellum tissue samples were from neonatal (postnatal day 1, P1), postnatal (P10), and puberal (P21) female mice (*Mus musculus*; *n* = 1 each age considered). Fixed cerebella were cut in blocks, frozen at -80°C and cryostat sectioned (30 μm thick) coronally.

### Gross Anatomy of Dolphin Tissue Slices

**FIGURE 3:**
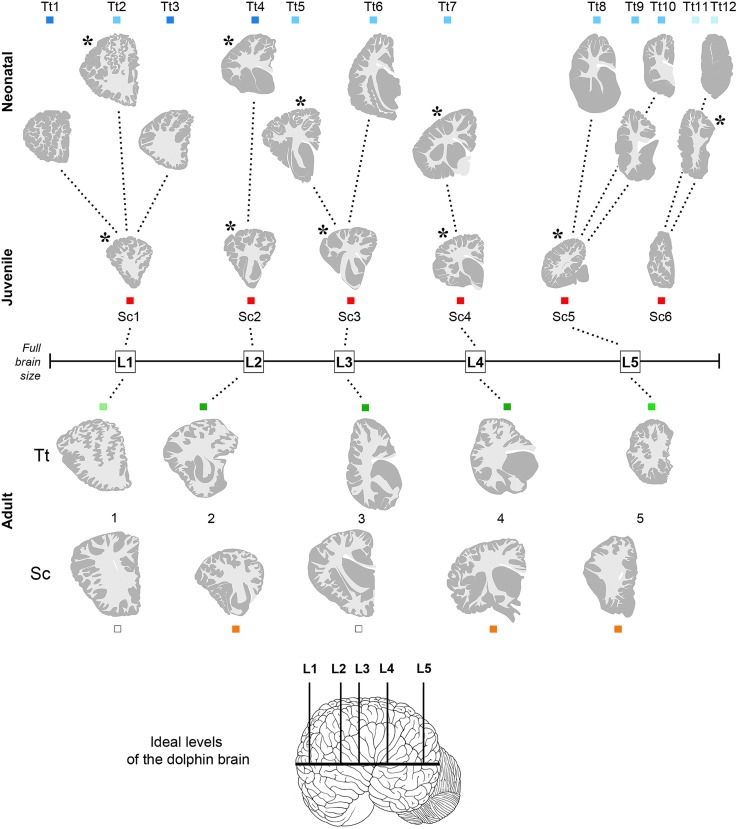
**Anterior–posterior levels obtained from drawings of coronal slices (as indicated in **Figure 2**) cut from brains of both species at different ages: neonatal *Tt*, levels Tt1–Tt12; juvenile *Sc*, levels Sc1–Sc6; adult *Tt* and *Sc*, levels L1–L5.** Squares of different colors indicate the animal specimens whose slices have been used for drawing single levels (the same as indicated in **Figure 1**). Note that more numerous, although less complete brains are available for neonatal *Tt*, whereas only one specimen, yet with a more complete brain, is available for juvenile *Sc*. All the young brains were used for establishing a forebrain atlas (see **Figure 7**). Adult brains were used for age comparisons and white/gray matter ratio quantification. The position of each level from left to right corresponds to its exact relative location with respect to the whole brain length. Dotted lines show the best matched coronal levels from adjacent slices; L1–L5, ideal levels shared by different animals, species and ages; asterisks indicate the levels closest to ideal levels. All drawings are in scale.

To obtain a graphic representation of individual brain levels, the front face of thick brain slices was photographed and imported in Neurolucida (Micro-Brightfield, Colchester,VT, USA), where the outlines of each coronal section, including those of the external (pial) surface and those at the white matter/gray matter limits, were drawn (**Figure 2A**). The contours were then imported in Photoshop to obtain images of each brain level (see **Figure 3**). This procedure was followed both for specimens that underwent histological analysis and for those that were simply photographed (see **Table 3**). These drawings were used for two types of analyses: (i) comparison of the neonatal/juvenile dolphin brain with previous literature data, such as MRI of similar age and/or atlases obtained from adult animals (**Figure 2C**); (ii) quantification of the WM/GM (see below).

### Tissue Processing for Histology, Gallyas, and Immunocytochemistry

Smaller blocks were cut from the thick formalin-fixed tissue slices (about 1,5 cm × 2,5 cm; see **Figure 2B**), washed in 0.1 M phosphate buffer (PB), pH 7.4, for 24 h, then cryoprotected sucrose solutions at gradually increasing concentrations, up to 30% in 0.1 M PB and subsequently frozen by immersion in liquid nitrogen-chilled isopentane at -80°C. Cryostat sections (40 μm thick) were cut on glass slides treated with 3-Aminopropyltriethoxysilane (Sigma–Aldrich, 741442) and processed for histological, silver staining, and immunocytochemical analyses. Some cryostat sections were processed for standard Gallyas stain of myelin using silver nitrate (Gallyas, 1979). Thick slices and blocks thereof used for histological analyses at different anterior–posterior levels and different ages are summarized in **Table 4.**

**Table 4:** Summary of all thick slices and relative blocks (see also **Figure 2B**) used for histologic analyses in the neonatal, juvenile, and adult dolphin brains, at different anterior–posterior levels (see **Figure 3**).

Species Age	ID	Brain level (slices)	Tissue blocks	
			
			Hist	Gal
*T. truncatus* (neonatal)	186 (L)	Tt2	1	1
		Tt5	3	2
		Tt7	3	1
		Tt9	1	1
		Tt10	1	–
	186 (R)	Tt6	1	1
		Tt10	2	–
	145 (L)	Tt3	2	–
		Tt4	2	1
		Tt8	1	–
		Tt9	1	–
		Tt11	1	–
	144 (L)	Tt4	2	–
		Tt5	2	1
		Tt6	1	–
		Tt7	1	–
		Tt10	1	–
	144 (R)	Tt4	2	–
		Tt5	2	–
		Tt6	1	–
		Tt7	1	–
		Tt10	1	–
	229 (L)	Tt9	1	–
		Tt12	–	–
*S. coeruleoalba*(juvenile)	320 (L)	Sc1	–	–
		Sc2	1	–
		Sc3	1	1
		Sc4	1	1
		Sc5	1	1
		Sc6	1	–
	320 (R)	Sc3	1	1
		Sc4	–	–
*Tt* (adult)	192 (L)	aTt2	–	–
		aTt3	1	1
		aTt4	1	1
	196 (L)	aTt3	1	1
		aTt4	–	–
*Sc* (adult)	167 (L)	aSc2	–	–
		aSc3	2	1
		aSc4	1	1
		aSc5	1	–


*ID, Animal identification number; L, left hemisphere, R, right hemisphere; Hist, histology; Gal, Gallyas.*

For immunocytochemical analyses, the sections were incubated in 1% H_2_O_2_ – phosphate-buffered saline (PBS) for 20 min, rinsed in PBS and then pre-incubated for 1 h at room temperature in blocking buffer [3% horse serum (HS), 2% bovine serum albumin (BSA), 1% Triton X-100 in 0.01 M PBS, pH 7.4] to reduce non-specific staining. The sections were then incubated for 24–48 h at 4°C in a solution of 0.01 M PBS, pH 7.4, containing 0.5% Triton X-100, 2% HS, 1% BSA and a primary antibody directed against Ki67 (NCL-Ki67p, made in rabbit, Novocastra, 1:1000), a nuclear protein expressed in all phases of the cell cycle except the resting phase, commonly used as a marker for cell proliferation; Glial Fibrillary Acidic Protein (GFAP, made in rabbit, Dako, 1:2000), an astrocytic marker. Immunohistochemical reactions were performed by the avidin–biotin–peroxidase method (Vectastain ABC Elite kit; Vector Laboratories, Burlingame, CA, USA) and revealed using 3,3′-diaminobenzidine (3% in Tris-HCl) as chromogen. The sections were counterstained with Cresyl violet, according to standard procedures currently employed in our lab at the N.I.C.O. (see Ponti et al., 2006a,b), mounted with DPX Mountant (Sigma–Aldrich, 06522) and examined using an E-800 Nikon microscope (Nikon, Melville, NY, USA) connected to a color CCD Camera.

### Image Processing and Data Analyses

All the images were analyzed using Adobe Photoshop CS4 (Adobe Systems, San Jose, CA, USA). Only general adjustments to color, contrast, and brightness were made.

Quantitative evaluations (cell density, cell size, WM/GM rate) were performed by means of the Neurolucida software (MicroBrightfield, Colchester, VT, USA). Measurements were obtained from: 20 sections in total from four animals (two animals for each ages – neonatal/juvenile and adult) to determine cell density (expressed as number of cells/area); 200 cells in total from four animals (two animals for each age), to determine cells size (area); 116 photographed slices from 13 animals (*n* = 5 neonatal, *n* = 1 juvenile, hereafter merged and referred to as neo/juvenile; *n* = 7 for adult) for WM/GM measurements (see **Table 5**). To compare different animals, regions of interest with matching positions along the anterior–posterior axis were chosen (**Figure 3**). All the graphs were obtained using Graph Pad Prism (San Diego California, USA). Statistical analyses were performed by Graph Pad Prism software and included unpaired (two-tailed) Student’s *t*-test (comparing only two groups). *p* < 0.05 was considered as statistically significant. Data are expressed as mean ± SD.

**Table 5:** Material employed for analysis of white/gray matter ratio (W/G) in the dolphin brain at different ages. Each level corresponds to a photographed brain slice.

Age	Species	ID	Representative brain levels				
			
			L1	L2	L3	L4	L5
*Neonatal*	*Tt*	Tt-144	Tt 3 (L)	Tt 4 (L/R)			
		Tt-229					Tt 9 (L)
							Tt 11(L)
		Tt-145	Tt 3 (L/R)	Tt 4 (L/R)	Tt 5 (L/R)		
		Tt-186	Tt 2 (L/R)		Tt 5 (L/R)	Tt 7 (L)	Tt 9 (L)
					Tt 6 (L)	Tt 8 (L)	
		Tt-162		Tt4(L/R)			
*Juvenile*	*Sc*	Sc-320	Sc 1 (L/R)	Sc 2 (L/R)	Sc 3 (L/R)	Sc 4 (L/R)	Sc 5 (L/R)
*Adult*	*Tt*	Tt-192	Tt 1 (L/R)	Tt 3 (L/R)		Tt 9 (R)	Tt 10 (L/R)
		Tt-196	Tt 1 (L)	Tt 3 (L/R)	Tt 5 (L/R)	Tt 7 (L/R)	Tt 9 (R)
					Tt 6 (L)	Tt 8 (L/R)	
		Tt-203	Tt 1 (L/R)	Tt 3 (L)	Tt 3 (R)	Tt 7 (R)	
			Tt 2 (L/R)				
		Tt-319	Tt 1 (L)	Tt 4 (L/R)	Tt 5 (L/R)	Tt 7 (L/R)	Tt 10 (R)
			Tt 2 (L/R)		Tt 6 (L/R)	Tt 8 (L/R)	Tt 11 (R)
			Tt 3 (L/R)				
		Tt-344	Tt 1 (L/R)	Tt 4 (L/R)	Tt 5 (L/R)	Tt 7 (L/R)	Tt 10 (L/R)
			Tt 2 (L/R)		Tt 6 (L/R)	Tt 8 (L/R)	Tt 11 (L/R)
			Tt 3 (L/R)				
	*Sc*	Sc-167	Sc 2 (L/R)	Sc 4 (L)	Sc 5 (L/R)	Sc 7 (L/R)	Sc 9 (L/R)
		Sc-218	Sc 2 (L)	Sc 4 (L)	Sc 5 (R)	Sc7 (L/R)	Sc 10 (L)
			Sc 3 (L/R)	Sc 5 (L)	Sc 6 (L/R)	Sc 8 (L/R)	
						Sc 9 (L)	


### Identification of Main White and Gray Matter Profiles in Brain Tissue Slices from Neonatal Bottlenose Dolphins, and Adult Bottlenose and Striped Dolphins: Definition of Brain Levels

Anterior–posterior levels were obtained by considering the drawings derived from all slices examined in young animals (see **Figure 1** and **Table 3**): a total of 12 (T1–T12, for neonatal *Tt*) and 6 (S1–S6, for juvenile *Sc*) (**Figure 3**). The higher number of levels in *Tt* was due to greater availability of animal specimens with respect to *Sc* (see also **Figure 1**). Five ideal levels of the dolphin brain were identified by adding and comparing all brain levels of *Tt* and *Sc*, so that each of the actual levels (and slices) analyzed in our study could be related to one of them (**Figure 3**). For instance, analyses were carried out on all the 12 levels to better exploit the *Tt* material, then the conclusions were centered on those levels which appeared more similar to the six *Sc* levels and to the five ideal levels (correspondences are indicated in **Figure 3**). Slices from adult animals (both species) were drawn in the same way. These drawings were intended mainly for comparative assessment of WM/GM; they were also used to assemble the atlas, in addition to literature data (The Yakovlev-Haleem collection at the National Museum of Health and Medicine; Pilleri et al., 1980).

## Results

### Cytoarchitecture in Major Subcortical Structures of Neonatal Bottlenose Dolphins and Juvenile Striped Dolphins: an Atlas of the Neonatal/Juvenile Dolphin Forebrain

**FIGURE 4:**
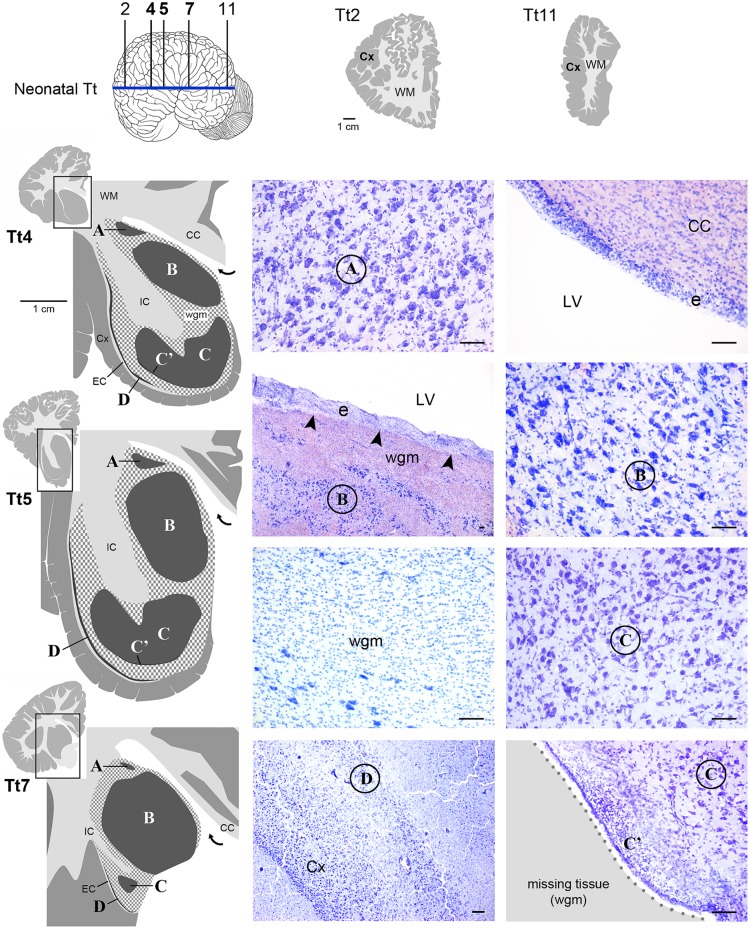
**Histological identification of cytoarchitectonics in the forebrain of the neonatal *Tt*.** Four main nuclei can be identified in the forebrain subcortical gray matter (A–D), indicated with Times New Roman font and circled on photographs at representative coronal levels of the brain (Tt4, Tt5, Tt7; selected among those indicated in **Figure 3** and indicated top left). anteriorly and posteriorly to levels Tt2 and Tt11, respectively, no subcortical gray matter is present (top right). Dark gray, gray matter; light gray, white matter (WM); wgm, subcortical white matter intermixed with gray matter (checked pattern); Cx, cerebral cortex; CC, corpus callosum; EC, external capsule; IC, internal capsule; LV, lateral ventricle (bent arrow in enlarged drawings on the left); e, ependyma. Note that nucleus B and ependyma of the lateral ventricle wall are separated by a mixture of white and gray matter in the absence of any thick, tightly packed cell layer which is typical of germinal layers in neonatal terrestrial mammals (arrowheads; see text). Scale bars, 100 μm.

**FIGURE 5:**
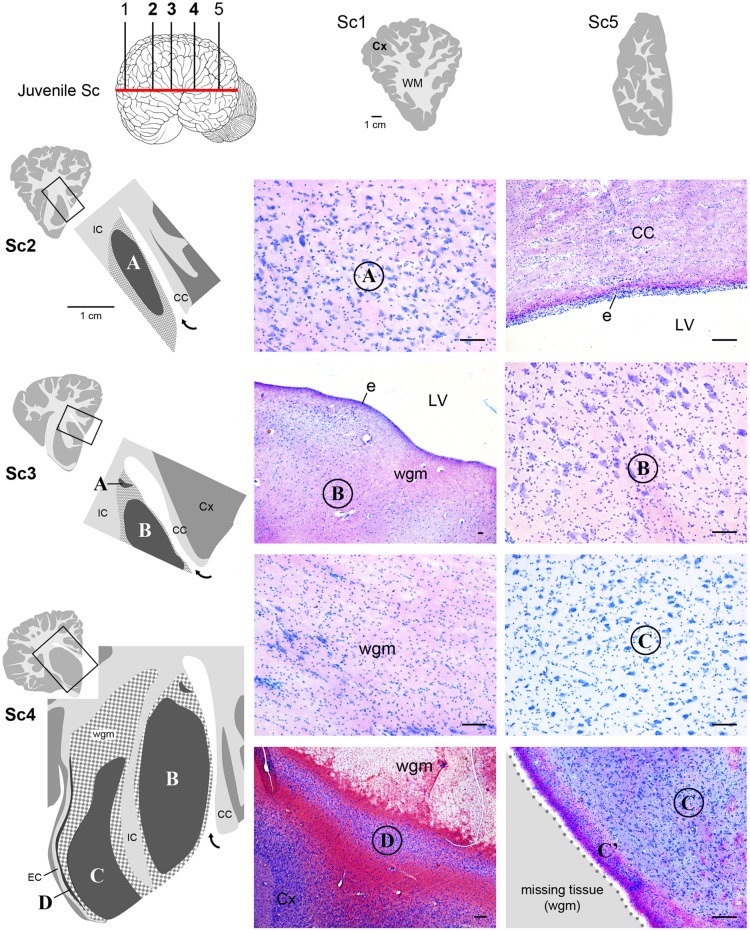
**Histological identification of cytoarchitectonics in the forebrain of the juvenile *Sc*.** No subcortical gray matter is present anteriorly and posteriorly to levels Sc1 and Sc5, respectively. The same forebrain nuclei showed in **Figure 4** for neonatal *Tt* (A–D) Times New Roman font and circled on photographs are detectable in juvenile *Sc* at representative levels of the brain (*Sc*2–4). The enlarged drawing depicting Sc4 has been rotated with respect to the square for reasons of space. For abbreviations, see **Figure 4.** Scale bars, 100 μm.

**FIGURE 6:**
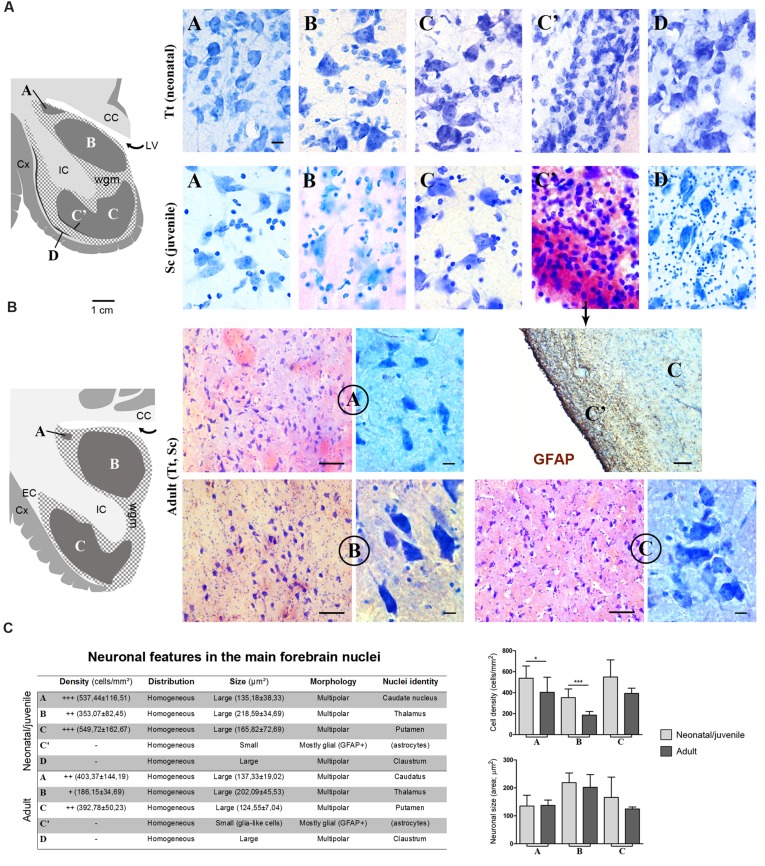
**(A)** Cytoarchitectonics and morphology of neurons in different forebrain nuclei of neonatal *Tt* and juvenile *Sc*. Gray matter nuclei are indicated in Times New Roman font (A–D), as in **Figures 4** and **5.** CC, corpus callosum; IC, internal capsule; LV, lateral ventricle; wgm, subcortical white matter intermixed with gray matter; Cx, cerebral cortex. Scale bar, 10 μm. **(B)** Histological analyses carried out on the adult (*Tt* and *Sc*) dolphin forebrain. The same forebrain nuclei (A–C) described in neonatal and juvenile dolphin brain can easily be identified in the adult forebrain of both species; abbreviations as in **(A)**. Scale bars: lower magnifications and GFAP staining, 100 μm; higher magnifications, 10 μm. **(C)** Table summarizing the main histological, morphological and quantitative measure parameters analyzed in the neonatal, juvenile, adult dolphin forebrain, leading to nuclei identification (also based on data reported in **Figures 4** and **5**); right, quantitative analyses of cell density and neuronal size in three forebrain nuclei at different ages.

**FIGURE 7:**
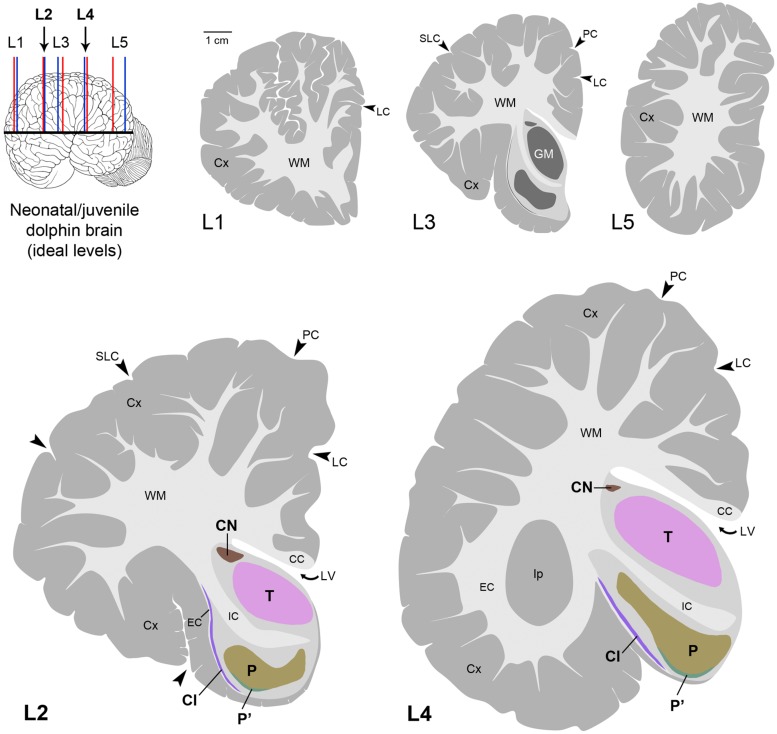
**Atlas in five levels of the ideal dolphin brain at perinatal stages, with particular reference to forebrain, subcortical periventricular structures (levels 2 and 4), based on the analyses of neonatal *Tt* and juvenile *Sc* reported in **Figures 4–6**; top left, blue and red lines indicate levels analyzed in neonatal *Tt* and juvenile *Sc*, respectively, then merged in five ideal levels (L1–L5, corresponding to those indicated in **Figure 3**).** CN, caudatus; P, putamen; T, thalamus; Cl, claustrum; LV, lateral ventricle; CC, corpus callosum; IC, internal capsule; EC, external capsule; LC, limbic cleft; SLC, superior lateral cleft; PC, paralimbic cleft; Ip, insular pocket. The amygdaloid complex was not considered.

Histological analyses based on Cresyl violet staining carried out at different anterior–posterior levels of forebrain regions (**Table 4**; **Figures 4** and **5**) were aimed at studying the cytoarchitectonics and neuronal morphology of the subcortical gray matter of the forebrain. Four major nuclei were identified both in neonatal *T. truncatus* and juvenile *S. coeruloalba* (indicated with A–D in capital Times New Roman font in **Figures 4**–**6**). These nuclei frequently had ill-defined limits, since they were separated by a combination of white and gray matter, well recognizable with respect to homogeneous white matter tracts (e.g., the corpus callosum and the internal capsule). However their extension and neuroanatomical location were very similar in neonatal and juvenile dolphins of both species. A five level (L1–L5) atlas was drawn by comparing the gross anatomy of the brain slices and forebrain histology/cytoarchitecture obtained from our series of neonatal *T. truncatus* and juvenile *S. coeruloalba* with previous MRI data obtained in young animals, and histological atlas of the adult dolphin brain (The Yakovlev-Haleem collection at the National Museum of Health and Medicine; The Welker collection at the University of Wisconsin Madison). For *S coeruloalba*, in the absence of species-specific reference work, we used a MRI study (Marino et al., 2004b) performed on *Sl*, that belongs to the same genre. Special attention was given to levels L2 and L4, that host most of the forebrain nuclei (**Figure 7**). Given the aim of the present study, we focused on forebrain areas directly surrounding the lateral ventricle and possibly hosting remnants of the germinal layer of the embryonic subventricular zone. The amygdaloid complex was not considered in detail. Comparative analyses of these nuclei in young and adult dolphins showed striking similarities in area and topography, in line with the advanced development of the dolphin brain in the perinatal phase. To verify such conclusion, qualitative and quantitative analysis were performed at different ages (**Figure 6**). All neurons, at all ages examined, showed a prevalent multipolar morphology and constant size of the cell body (**Figure 6C**). The lateral part of nucleus C was characterized by small, densely packed cells (C′), subsequently identified as glial cells by immunocytochemical staining for the astrocytic marker GFAP (showed only in adults in **Figure 6**). In addition, neuronal cell density and size were evaluated in three forebrain nuclei (A, B, C) at different ages (**Figure 6C**). The average cell density was higher in some nuclei of the neonate (A and B) with respect to adults (A, ^∗^*p* = 0,0340; B, ^∗∗∗^*p* < 0,0001). On the whole, histological, morphological and quantitative data clearly identified the caudate nucleus (A), thalamus (B), putamen (C), and claustrum (D) (see Table in **Figure 6C**, and atlas in **Figure 7**), and confirmed substantial similarities between neonatal and adult brains.

Interestingly enough, either the caudate nucleus (A) and thalamus (B), or, more frequently, an intermixed white/gray matter stripe, were lying directly beneath the ependyma (**Figures 4** and **5**), all along the lateral wall of the lateral ventricle (extending from levels Tt4 to Tt10 and Sc2 to Sc5). Therefore the thick germinal layer typically occurring in the periventricular region of all neonatal terrestrial mammals studied so far was clearly absent (see Discussion for references). Further immunocytochemical analysis of Ki67 antigen expression in the lateral wall of the lateral ventricle did not detect any proliferative activity in periventricular, sub-ependymal position (not shown), thus confirming the absence of periventricular germinal layers in dolphins around birth.

### White Matter Quantification in the Neonatal, Juvenile, and Adult Dolphin Brain

**FIGURE 8:**
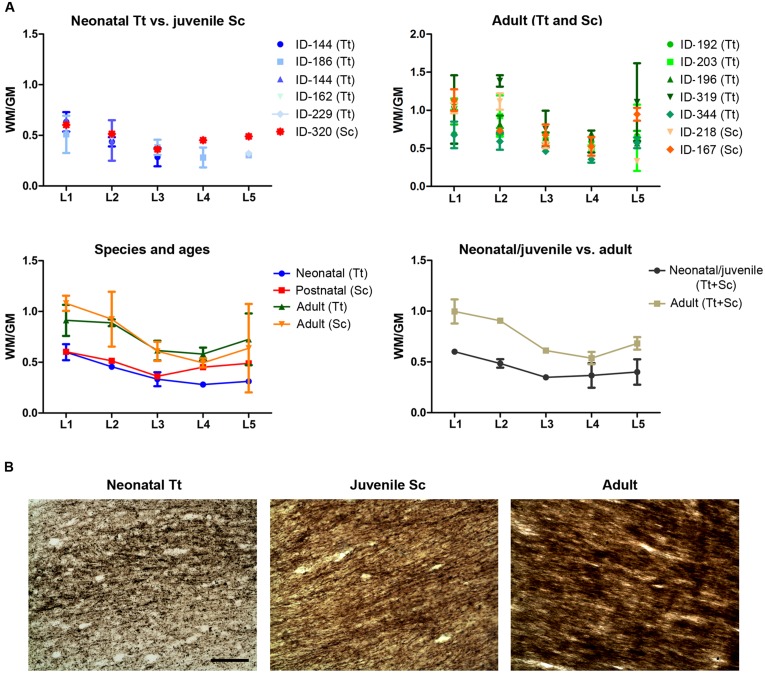
**White matter development in dolphins of different ages.** **(A)** Analysis of white/gray matter ratio (W/G) in the neonatal, juvenile, and adult dolphin (neonatal *Tt*, juvenile *Sc*, adult *Tt* and *Sc*). **(B)** Myelinization in axons of the corpus callosum detected with Gallyas stain technique. Scale bar, 100 μm.

A quantification of the areas was performed to measure the WM/GM ratio by using the drawings of white and gray matter limits in each coronal slice cut at different levels (approximated to standard brain levels, L1–L5) in 13 animals of both species and different ages (**Table 5** and **Figure 8**). On the whole, the WM/GM ratio was lower in neonatal (0,39601 ± 0,13137) and juvenile (0,48419 ± 0,088024) individuals (**Figure 8A**, top left), whereas it was higher (0,73267 ± 0,19124) in adults (**Figure 8A**, top right). Statistical analysis was performed by adding all brain levels available in all animals, since in some animals it was not possible to have all levels. Comparison of neonatal *Tt* (0,3960 ± 0,1314) vs. juvenile *Sc* (0,4842 ± 0,0880), and juvenile (0,4842 ± 0,0880) vs. adult (0,7401 ± 0,1860), showed no significant differences (*p* = 0,2477 and ^∗^*p* = 0,0239, respectively; unpaired *t*-test – two tailed). However, a small but significant difference (^∗^*p* = 0,015; unpaired *t*-test – two tailed) was detected between the neonatal/juvenile (0,4401 ± 0,1042) and adult brains (0.7469 ± 0,1964) (**Figure 8A**, bottom). Standard deviations were higher in some adult specimens, particularly for levels L1, L2, L5, namely, the most rostral or posterior parts, where the white matter is progressively replaced by cortical gray matter.

The extent of myelination was assessed by Gallyas stain. Such analysis revealed the presence of myelin sheaths in the corpus callosum of dolphins already around birth, the pattern of stain being only slightly increased in intensity at juvenile and adult ages (**Figure 8B**).

### Extension/Exhaustion of Transient Germinal Layers in the Cerebellar Cortex of The Neonatal *T. truncatus* and Juvenile *S. coeruloalba* (and Comparison with Mouse)

**FIGURE 9:**
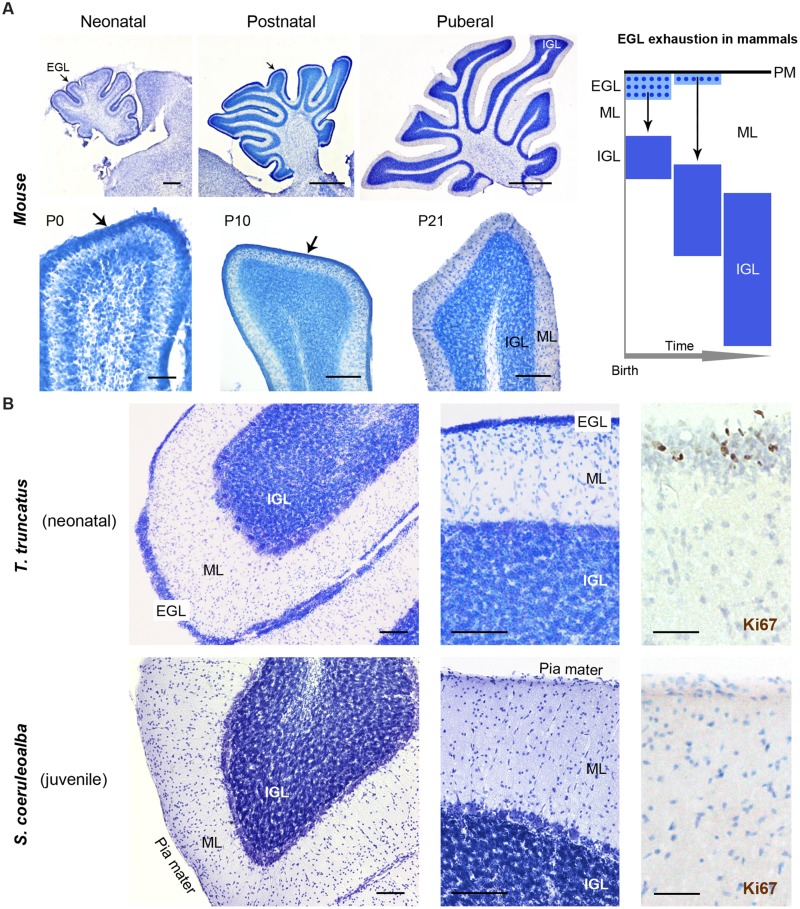
**Histological study and cell proliferation analysis in the cerebellar cortex of mice and dolphins, at different postnatal ages.** **(A)** Mouse cerebellum at neonatal, postnatal and puberal stages; the panel on the right summarizes the morphogenesis of the inner granule layer (IGL) in mammals, which occurs mostly postnatally through migration of granule cell precursors from a subpial, proliferative external granule layer (EGL, subsequently exhausted). Micrographs: note the absence of well formed IGL and molecular layer (ML) in the mouse cerebellar cortex at birth, and the persistence of a proliferative EGL until the pre-puberal ages; at puberal stages the IGL is fully formed and the EGL is exhausted. **(B)** Dolphin cerebellar cortex at neonatal (top) and juvenile (bottom) stages. Note the thick, well formed IGL and ML in *T. truncatus* at birth; a thin, proliferative EGL (Ki67+ cells on the right) is still present on the cerebellar surface. In juvenile *S. coeruloalba* (bottom) the EGL has disappeared and the ML is directly in contact with the cerebellar surface (pia mater). Scale bars: 100 μm; mouse low magnifications (top), 500 μm; mouse P0 and Ki67, 50 μm.

Histological analysis (Cresyl violet staining) and Ki67 antigen immunocytochemical detection were used to investigate the existence and/or exhaustion of a proliferative external granule layer (EGL; a hallmark of cerebellar development in mammals; **Figure 9A**, top right) in the cerebellar cortex of neonatal *Tt* and juvenile *Sc*. For comparison we also analyzed neonatal, postnatal, and adult mouse cerebella (**Figure 9A**). Laboratory rodents have a thick EGL at birth, whereas a clear-cut molecular layer (ML) and a well formed inner granular layer (IGL) are lacking (**Figure 9A**, top). The entire granule cell population of IGL completes its formation only later, at pre-pubertal stages, after progressive thickening of both ML and IGL during postnatal stages (**Figure 9A**, bottom).

The cerebellum of neonatal *Tt* showed a relatively thin EGL in subpial position (recognizable as a layer of tightly packed, small, darkly stained cells with numerous actively proliferating cells) on the cerebellar surface, and thick, well formed IGL and ML within the cerebellar cortex (**Figure 9B**, top; **Table 6**). Immunodetection of Ki67 antigen clearly confirmed the occurrence of cell proliferation in the EGL of neonatal *Tt* (**Figure 9B**, top right). By contrast, no histological evidence for EGL was detectable in the juvenile *Sc* cerebellar cortex (**Figure 9B**, bottom), whose subpial surface, as expected, was also devoid of proliferating cells, thus showing that exhaustion of proliferative EGL has already occurred in dolphins 3–6 months after birth. However, the extension of IGL in juvenile *Sc* was quite similar to that observed in neonatal *Tt*, supporting the evidence for an advanced stage of development in the cerebellar cortex of the dolphin around birth (see **Table 6**).

**Table 6:** Existence/exhaustion of transient germinal layers and formation of mature neuronal layers in the cerebellar cortex of neonatal *Tt*, juvenile *Sc*, and neonatal, postnatal, pubertal mouse.

Species	ID	Age	EGL		IGL	ML
					
			Presence	Ki67+		
*T. truncatus*	144	Neo	Yes	Yes	Almost formed	Thick, sharp
	145					
	186					
	229					
*S. coeruleoalba*	320	J	No	No	Fully formed	Thick, sharp
*Mouse*	M-1	Neo	Yes	Yes	Not formed	Ill-defined
	M-2	P	Yes	Yes	Almost formed	Thin, sharp
	M-3	Pub	No	No	Fully formed	Thick, sharp


*Neo, neonatal (*Tt*, P9; mouse, P1); J, juvenile (*Sc*, P90–P180); P, postnatal (mouse, P10); Pub, pubertal (mouse, P21). EGL, External granule layer; IGL, inner granule layer; ML, molecular layer.*

## Discussion

In the present report we exploited material belonging to 10 bottlenose dolphins and three striped dolphins conserved at the MMMTB in Padova, with the aim of gaining a deeper insight into the neuroanatomy of the neonatal/juvenile dolphin brain. We focused on developmental stages during the perinatal period, with particular reference to forebrain and cerebellar regions that are known to host neurogenic layers in terrestrial mammals. The development of dolphins has been studied and described in detail in the accurate work of Štěrba et al. (2000). In their comprehensive article, Štěrba et al. (2000) analyzed several specimens of *Sl*, *Sa*, *Dd*, and *Phocoena* (this latter non-delphinid species is not relevant here and will not be discussed further). The specimens were painstakingly compared to the reference stages detailed by O’Rahilly (1972), who used embryonic and fetal material from the Carnegie reference collection. Since marine mammals (including dolphins) lack external hair (a key temporal landmark for developmental stages in terrestrial mammals), Štěrba et al. (2000) established a new set of stages adapted from that of O’Rahilly. Data relative to staging and development basically demonstrated that (not-surprisingly) the specimens belonging to the genus *Stenella* and *Delphinus* are very similar. The body measures of all the species fall within the same range, with minimal variations due to the single specimens and their relative sample numerosity in the different stages. Another study on the same subject (Thewissen and Heyning, 2007) adopted a different staging for fetal development in dolphins, but came to conclusions very similar to those of Štěrba et al. (2000).

The rationale for comparing the species that we mainly describe in our study, i.e., the bottlenose dolphin, with specimens of the striped dolphin, is based on their common origin in the family *Delphinidae* and on the similarity of their morphology (see also **Table 2**). Furthermore, their developmental stages are comparable, as showed in a recent study (Cozzi et al., 2015) in which embryos and fetuses of *Sc*, *Dd*, and *Tt* were analyzed for progressive very early deposition of calcium salts in the tympanoperiotic complex (a rather distinctive trait of marine Cetartiodactyla). The bottlenose dolphin *Tt* is approx. 1/10 larger than *Sc* at birth in the Mediterranean area (Cagnolaro et al., 2015), but the stages of fetal development are very similar and follow the same pattern.

**FIGURE 10:**
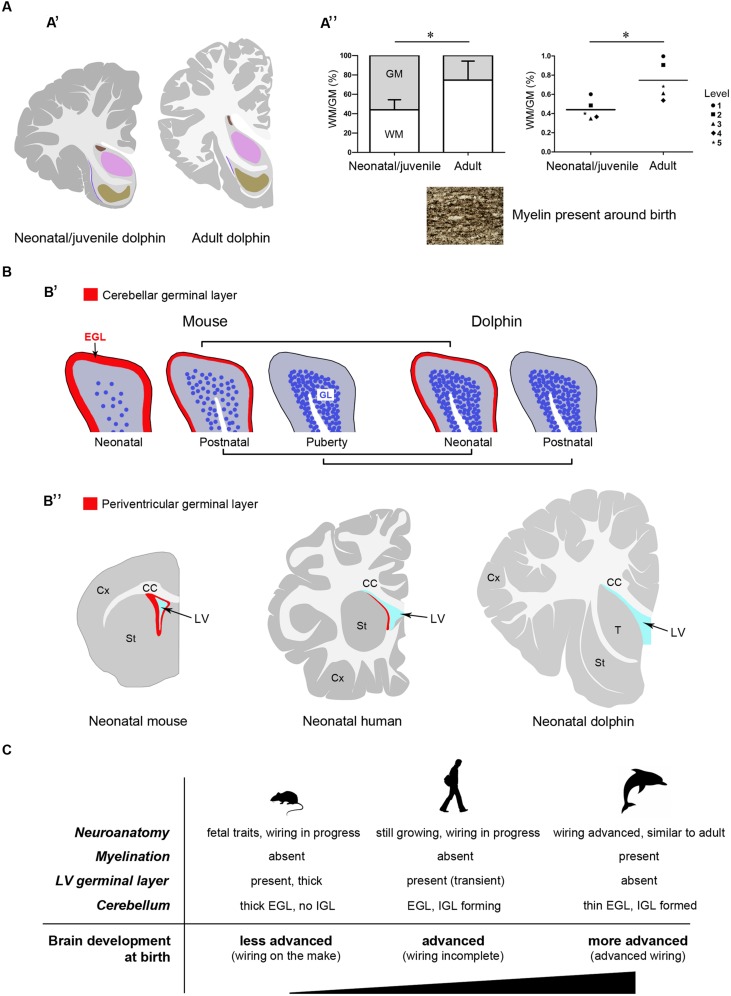
**Comparison between neonate and adult dolphins, and between dolphins and terrestrial mammals.** Different features converge to confirm the advanced developmental stage of the dolphin nervous system at birth. **(A)** Neuroanatomical features: **(A’)**, neonatal/juvenile dolphin brain anatomy is very similar to that described in adults; **(A”)** white matter growth and myelination in dolphins are already advanced at neonatal/juvenile stages; WM, white matter; GM, gray matter; in the graph on the right the means calculated at single brain levels of different animals are showed. **(B)** Existence/exhaustion of postnatal germinal layers; **(B’)**, advanced development of the cerebellar cortex in neo/postnatal dolphins: the external germinal layer (EGL) is extremely thin at birth and disappears at a very juvenile stage; the granule cell layer (IGL) is almost formed at birth; **(B”)** no germinal layer is detectable along the wall of the lateral ventricle at birth in dolphins, whereas it is present in all mammals studied so far (see references in the text). **(C)** The neonatal brain: comparison between aquatic and terrestrial mammals.

Here we present an atlas of the neonatal/juvenile forebrain regions (**Figure 7**). This allowed comparing the neuroanatomical features observed in the very young dolphin with data gathered in our adult specimens, and with data currently available in the literature for different species of adult dolphins (The Yakovlev-Haleem collection at the National Museum of Health and Medicine; The Welker collection at the University of Wisconsin Madison; Jacobs et al., 1979; Pilleri et al., 1980; Oelschläger et al., 2008; see **Figure 2C**). The four main characteristics of brain morphology investigated in our study (i–iv in the following text; summarized in **Figure 10**) indicate an advanced developmental stage of the dolphin brain at birth, and were derived from separate but convergent neuroanatomical observations: (i) forebrain neuroanatomy; (ii) perinatal myelination; (iii) postnatal genesis of granules in the cerebellar cortex; (iv) absence of a well defined sub-ventricular germinal layer. Though comparisons of two different species, even those that are morphologically similar, carries the potential for misinterpretations of results, our data shows that the brains of the bottlenose and striped dolphin are easily comparable. Although their absolute weights differ, the sizes of the single components (selected nuclei of the forebrain, cortical areas, etc.) remained proportionally consistent throughout the experimental series.

### (i) Forebrain Neuroanatomy

Topographical location, extension, and cytoarchitectonics of the main forebrain nuclei are very similar at different ages from birth to adulthood (**Figures 4–6**). The aspect of corresponding coronal slices (e.g., **Figure 10A’**), and the neuronal morphology and size in the main forebrain nuclei appear quite similar at all ages (**Figure 6**). A slightly increased neuronal density in the caudate nucleus and thalamus around birth could simply depend on the growth in size of these nuclei (a similar trend was previously reported for cortical areas in the brain of the same species; Garey and Leuba, 1986). These observations suggest early maturation of the basal forebrain circuits, including the striate and its connections, which are fundamental for motor coordination and learning abilities (Ridgway, 1990). In humans, the brain grows from just under 25% of its adult weight at birth to full development with complete wiring by approximately 17–18 years. Such prolonged dependence on parents increases the period during which learning can occur (Bonner, 1980; Ridgway, 1990). Dolphins are born after a long gestational period (about 12 months, see **Table 2**) with brains at an advanced stage of development, which enables them to swim and perform activities (including decodification of acoustic signals essential for orientation and familial bonding) requiring a relative maturity. It has been estimated that the weight of the dolphin brain at birth is about 42% of the average weight of the adult brain, and exceeds 80% at weaning (18 months), which is higher than the value obtained for the human brain at 3–4 years of age. Dolphins are considered to reach full brain development when they reach sexual maturity (see **Table 2**), i.e., in about half the time required for the development of the human brain (see also **Figure 10C**).

### (ii) White Matter Growth and Myelination Around Birth

In our experimental series, the growth of white matter is well advanced very close to birth and myelination has already occurred (**Figure 10A”**). As previously described in studies using non-invasive approaches in the Atlantic white-sided dolphin (a species member of the same dolphin family, see **Table 2**), white matter measurements in MRI represent myelinated or mature white matter volumes, whereas gray matter measurements are the sum of immature white matter, cortical gray matter, and subcortical gray matter volumes (Montie et al., 2008). Thus, white matter in neonates can be underestimated by MRI (at least in Atlantic white-sided dolphin, another species of the family *Delphinidae*), whereas in our specimens white and gray matter were identified directly on the slices. We only found a slight increase in the WM/GM from neonates to adults (**Figures 3** and **10A”**), thus confirming that white matter growth is already advanced around birth, which has also been suggested by previously reported biochemical studies (Lesch, 1969). The occurrence of myelination was also investigated through histological analysis with Gallyas staining, which confirmed its presence around birth. Myelination is nearly complete at birth in precocious animals; in most mammals it occurs relatively late in development in a defined temporal sequence. In mice and rats, it starts at birth in the spinal cord but the brain is involved only later in postnatal life, whereby most of the nerve fibers in the corpus callosum are non-myelinated in the neonate (Sternberger et al., 1978; Vincze et al., 2008). In humans, the peak of myelination occurs much later, during the 1st year of life, and coincides with the development of motor abilities and specific cognitive functions (Fields, 2008; Snaidero and Simons, 2014). On the other hand, newborn terrestrial Cetartiodactyla (including ruminants, swine, camels) must be immediately able to stand and move to survive in the wild. The advanced myelination at birth that we note here in dolphins (marine Cetartiodactyla) fulfills the immediate need of the newborn to possess all the swimming competences required for life, including the ability to reach the surface and breath.

### (iii) Postnatal Genesis of Granules in the Cerebellar Cortex

Analysis of the developmental stage of delayed neurogenic processes also converged to indicate advanced brain maturation. Indeed, the postnatal genesis of the population of granules in the dolphin cerebellar cortex is quite advanced. The cerebellar cortex is a hallmark of postnatal neuronal development in mammals, characterized by a process of delayed neurogenesis (see Ponti et al., 2008; Bonfanti and Peretto, 2011) leading to formation of the largest CNS neuronal population, i.e., the granule cells, organized in the cell IGL. The IGL derives from radial, centripetal migration of granule cell precursors of a proliferating, transient external granule layer or external germinal layer (EGL; Altman,1969; Abraham et al., 2001; **Figure 9A**). EGL exhaustion on the cerebellar surface occurs at species-specific postnatal stages and is tightly linked to postnatal CNS maturation in mammals that differ in their degrees of motor skills at birth, age of puberty, and lifespan (Sanchez-Villagra and Sultan, 2002; Ponti et al., 2008, 2010). In altricial mammals (somatically immature at birth, such as the mouse and rat), the formation/maturation of the cerebellum can be protracted until puberty, since this organ is crucially involved in the acquisition of coordinated sensorimotor behavior. Yet, across mammals, the most mature cerebella at birth still have an EGL, indicating that the mossy fiber-granule cell connectivity is not yet fully developed and may depend on external experiences to fully mature (Sanchez-Villagra and Sultan, 2002). Our analysis of the dolphin cerebellar cortex shows signs of very advanced development, i.e., substantial IGL formation close to birth, and EGL disappearing at very early juvenile stages (3–6 months) with respect to the age of puberty (around 5–7 years, or even later) (**Figure 10B”**).

### (iv) Absence of a Well Defined Sub-ventricular Germinal Layer

In our study, both histology and Ki67 antigen immunocytochemical detection did not reveal any proliferative activity in the sub-ependymal area of the lateral ventricle, suggesting the absence of periventricular germinal layers in dolphins at birth. Here we emphasize that the strong Ki67 antigen stain obtained in the EGL of the cerebellar cortex also represented an internal control (**Figure 9B**).

All the aspects examined here depend on several variables linked to the timeframe of pre- and post-natal development which include duration of the gestation period, independence of pups at birth, age of puberty, and animal lifespan, among others (see Workman et al., 2013 and the related site: http://www.translatingtime.net). It is therefore difficult to identify specific inferences, rather than a general trend. Our conclusions concerning the developmental stage of the neonatal dolphin brain are intended to stay within limits of a confirmation of previous findings, the main focus of this study being the neonatal dolphin forebrain in a perspective of future analyses of neurogenic processes. In this context, data obtained on dolphin cerebellar maturation fit well with the apparent lack of periventricular germinal layer in the neonatal forebrain. The absence of a subventricular zone-derived germinal layer in neonatal dolphins appears to set them strikingly apart from all terrestrial mammals studied so far, including rodents and humans (Tramontin et al., 2003; Peretto et al., 2005; Sanai et al., 2011; **Figure 10B’**). In humans, whose brain size and lifespan match those of most dolphins species, such periventricular layer is still detectable at birth (Del Bigio, 2011; Sanai et al., 2011), although almost exhausted by 18 months of postnatal life (Sanai et al., 2011; Wang et al., 2011). Its absence in neonatal dolphins, besides confirming the advanced developmental stage of their brain at birth, might open the possibility that persistent forebrain neurogenesis, which is known to supply new neurons to the olfactory bulb in all mammals studied so far (Lois and Alvarez-Buylla, 1994), might not persist in marine mammals devoid of olfaction/olfactory brain structures. Although dolphins and other toothed whales possess a terminal nerve and its relative ganglion (Oelschläger et al., 1987; Demski et al., 1990), they remain in the primitive meninx and their function is still uncertain (Buhl and Oelschläger, 1986). A recent study carried out in the cetacean hippocampus by doublecortin detection reported absence of this marker of structural plasticity in the dentate gyrus, thus suggesting that adult hippocampal neurogenesis could be absent in aquatic Cetartiodactyla (Patzke et al., 2015). Based on these observations, if a residual germinal layer does persist within the forebrain of dolphins, it might be organized differently with respect to terrestrial mammals. Taking into account the extremely large size of the cetacean brain, the search for neurogenic remnants in these species may represent quite a titanic endeavor. The results obtained in the present study suggest that detailed investigation of the occurrence/absence/type of neurogenesis in aquatic Cetartiodactyla at birth (Parolisi et al., in preparation) could provide interesting insights into this fascinating topic, and set a neuroanatomical background for the achievement of this task.

## Conflict of Interest Statement

The authors declare that the research was conducted in the absence of any commercial or financial relationships that could be construed as a potential conflict of interest.
